# Personal Health Large Language Models and the Negotiation of Medical Authority in Clinical Care: Opportunities, Risks, and Governance

**DOI:** 10.2196/91727

**Published:** 2026-06-25

**Authors:** Wenyi Xie, Jialin Liu, Siru Liu

**Affiliations:** 1 Medical Intensive Care Unit, West China Hospital of Sichuan University, Chengdu, Sichuan, China; 2 Information Center, West China Hospital of Sichuan University, No. 37 Guo Xue Xiang Street, Chengdu, Sichuan, 610041, China, 86 28-85422416, 86 28-85582944; 3 Department of Otolaryngology-Head and Neck Surgery, West China Hospital of Sichuan University, Chengdu, Sichuan, China; 4 Department of Biomedical Informatics, Vanderbilt University Medical Center, Nashville, TN, United States

**Keywords:** personal health large language models, clinician-patient relationship, artificial intelligence, medical authority, clinical governance, shared decision-making, patient-generated health data, digital health, accountability

## Abstract

Personal health large language models (PH-LLMs) are patient-facing conversational systems that synthesize user-entered information, patient-generated health data, wearable data, and selected personal health records—where users choose to connect them—into personalized, longitudinal, action-oriented health narratives. Unlike generic health chatbots that mainly provide one-off responses to isolated questions, PH-LLMs may generate continuing interpretations, priorities, and candidate next steps that patients bring into clinical encounters. In contrast to electronic health record–tethered clinical artificial intelligence (AI), they often originate outside institutional oversight and may be selected, used, or trusted by patients before professional review. This viewpoint examines how PH-LLMs may reshape the negotiation of medical authority by contributing to a shift from the traditional dyadic clinician-patient relationship toward a triadic model of negotiated authority, in which clinicians may increasingly need to mediate among clinical evidence, patient values, and algorithmic narratives. PH-LLMs may support patient participation by organizing symptoms, contextualizing home-monitoring and wearable data, improving health literacy, assisting chronic disease self-management, and preparing patients for more collaborative visits. Patient-facing AI narratives may also introduce distinct risks. At the individual level, these include inaccurate or incomplete responses arising from imprecise queries or missing context, misinterpretation of otherwise accurate information in the absence of clinical context, and contextually biased or poorly matched advice across demographic, cultural, linguistic, disability-related, or socioeconomic contexts. At the system level, they include authority conflict when AI recommendations diverge from clinical judgment, fragmentation of clinical truth, privacy and data-governance concerns, diffusion of accountability when harm results from advice produced outside clinical governance, and inequitable access to premium tools and continuous monitoring devices. To address these challenges, we propose a 3-layer clinical governance framework for patient-brought PH-LLM narratives. The first layer, evidence and provenance, makes AI-generated narratives epistemically legible by clarifying platform identity, data sources, temporal anchoring, uncertainty, and privacy-relevant data-use and retention conditions. The second layer, clinical arbitration and workflow integration, uses risk-stratified intake, proportionate documentation, escalation triggers, and equity-preserving workflows to embed PH-LLM outputs into routine care. The third layer, competence and accountability, defines the communication competencies, AI literacy supports, institutional responsibilities, vendor accountability, and risk-proportionate verification duties needed for triadic care. This framework is a conceptual and governance-oriented proposal rather than a validated clinical protocol. Future empirical work should evaluate its feasibility, documentation burden, equity effects, clinical safety impact, and acceptability among patients, clinicians, and health systems. Governed through these interdependent layers, PH-LLMs may serve as supporting infrastructure for safer, person-centered longitudinal care.

## Introduction

In the conventional biomedical model, diagnostic reasoning and therapeutic recommendations have been formulated primarily within the clinical encounter, sustained by professional epistemic authority and institutional accountability [1]. This arrangement is increasingly challenged by the emergence of personal health large language models (PH-LLMs). In this viewpoint, PH-LLMs refer to patient-facing or consumer-facing large language model (LLM)-based conversational systems that synthesize user-entered information, patient-generated health data, wearable data, and selected user-connected personal health records into personalized, longitudinal, action-oriented health narratives [2-4]. This patient-initiated access to personal health records is distinct from bidirectional electronic health record (EHR) integration governed by health systems and clinical workflows. For clarity throughout this viewpoint, by epistemic authority we refer to the capacity of a knowledge source, including clinicians, clinical records, or PH-LLM outputs, to shape how users interpret symptoms, risks, and care options before or between clinical encounters. Compared with generic health chatbots, which mainly provide one-off responses to isolated questions, PH-LLMs may sustain continuing interpretations, priorities, and candidate next steps that patients carry into clinical encounters. They also differ from EHR-tethered clinical artificial intelligence (AI): whereas EHR-tethered AI generally operates within health-system governance and clinician workflows, PH-LLMs are typically initiated, selected, or used by patients before professional review [5]. We adopt this functional definition based on user-facing behavior and deployment context rather than on any specific model architecture, because PH-LLMs evolve rapidly and span both proprietary and open-source implementations.

Recent commercial developments illustrate this emerging category. Examples include OpenAI’s ChatGPT Health, which began rolling out in January 2026 as a dedicated health and wellness experience, and Fitbit’s Gemini-powered personal health coach, which entered public preview initially for eligible US-based Fitbit Premium Android users in October 2025 [6-10]. These products are referenced solely as time-bound illustrations of how consumer-facing PH-LLMs are entering everyday use, not as evidence of clinical decision-making capability, and their features, availability, and access conditions remain subject to change. Unlike static metric dashboards, PH-LLMs may synthesize wearable data, user-entered information, patient-generated health data, and selected personal health records—when users choose to connect them—into plausible explanations and candidate next steps before professional review or clinical contextualization [11]. However, such synthesis should not be equated with reliable longitudinal medical reasoning. Evidence from clinical AI deployment, longitudinal EHR reasoning, and LLM assurance studies suggests that current systems may remain vulnerable to data shift, temporal-reasoning limitations, and hallucination when interpreting noisy, incomplete, device-dependent, or temporally distributed health data [12-14].

**Figure 1.**
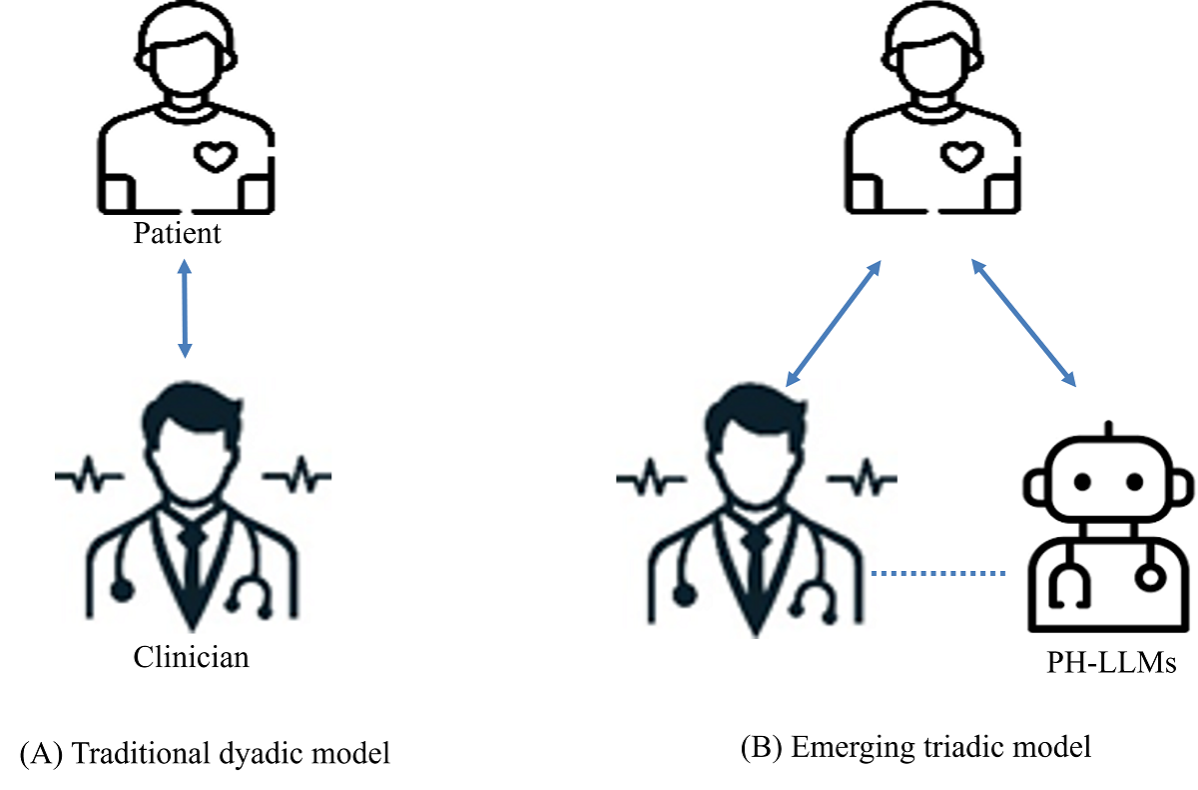
Transition from a dyadic clinician-patient relationship to a triadic clinician-patient-PH-LLM relationship. (A) The traditional dyadic model, in which clinical reasoning, risk interpretation, and treatment recommendations are negotiated primarily between the clinician and the patient. (B) The emerging triadic model, in which patients may use PH-LLMs outside institutional oversight, such as between visits or before encounters, and bring AI-generated summaries, interpretations, or recommendations into the visit. Solid lines indicate direct interactions between the patient and clinician, and between the patient and PH-LLM. The dotted blue line between the clinician and PH-LLM represents an indirect, patient-mediated relationship rather than direct interoperability, formal EHR integration, or clinician control. Clinicians therefore encounter PH-LLMs mainly through patient-brought outputs and must mediate among clinical evidence, patient values, and algorithmic narratives that may diverge from clinical judgment. This figure also illustrates that PH-LLMs may redistribute epistemic authority across 3 actors and thereby create a need for point-of-care governance mechanisms. AI: artificial intelligence; PH-LLM: personal health large language model.

PH-LLM functions should, therefore, be understood along a risk continuum. Low-risk functions include general health education, lifestyle coaching, sleep or fitness support, and visit preparation focused on question framing or symptom organization. Higher-risk functions include chronic disease management, medication-related advice, diagnostic interpretation, escalation guidance, or recommendations that conflict with clinician judgment. In practice, a single user session may span multiple points along this continuum, and the same functional category may carry different levels of risk depending on the patient population, clinical context, and the specificity of the AI-generated recommendation. As patients use these systems across this continuum, they may enter clinical encounters with AI-generated interpretations, priorities, and candidate next steps that prestructure clinical sensemaking. This may contribute to a shift from a dyadic model of clinician-patient interaction toward a triadic model of negotiated authority, in which clinicians may increasingly need to mediate among clinical evidence, patient values, and algorithmic narratives (Figure 1). While this potential shift may support chronic disease self-management and shared decision-making, it also introduces risk pathways, including epistemic conflict, fragmentation of clinical truth, privacy and data-governance concerns, and diffusion of accountability [2,15].

The purpose of this viewpoint is to analyze how PH-LLMs may reshape the negotiation of medical authority and propose a clinical governance framework that preserves safety, accountability, and trust while enabling beneficial use. This viewpoint focuses specifically on patient-facing PH-LLMs whose personalized outputs may enter clinical encounters and influence shared understanding, patient expectations, or clinical decisions; it does not address all health-related chatbots or all forms of clinical AI. We examine the potential implications of this emerging shift for clinical practice, characterize key opportunities and risks, identify recurrent failure modes, and outline governance strategies across 3 domains: (1) evidence and provenance, (2) clinical arbitration and workflow integration, and (3) competence and accountability.

This viewpoint is conceptual and governance-oriented. To avoid overstating the evidence base, we distinguish time-bound examples of consumer-facing deployment [6-10], emerging peer-reviewed evidence on PH-LLM synthesis of wearable and patient-generated data [11], evidence on capability limitations from clinical AI deployment, longitudinal EHR reasoning, and LLM assurance studies [12-14], and conceptual claims about how PH-LLMs may reshape patient interpretation, clinician-patient interaction, and accountability [2,16-20]. Projections of future clinical practice and governance outcomes are conditional on governance design, institutional adoption, and policy uptake rather than predictions of inevitable change. The proposed shift toward a triadic clinician-patient-PH-LLM relationship is therefore presented as an emerging governance concern rather than as an empirically established transformation. The observable markers in Table 1 are intended to support future empirical evaluation of these propositions, including their magnitude, distribution across populations, equity implications, and clinical safety effects.

**Table 1.** Sociotechnical concepts and candidate observable markers for the empirical evaluation of the integration of personal health large language models (PH-LLMs) in clinical encounters.

Sociotechnical concept	Candidate observable markers^a^
Triadic encounter and negotiated authority	Encounters in which patients introduce AI^b^-generated content, visit time allocated to AI-mediated discussion, and shared decisions in which AI advice is explicitly considered
Authority conflict	Documented AI-clinician disagreement; plan modification, follow-up, or escalation after disagreement; and patient-reported adherence changes after disagreement
Fragmentation of clinical truth	Multiple AI platforms or wearable systems referenced in one visit, conflicting AI-generated interpretations, and clinician time spent reconciling inconsistent narratives
Diffusion of accountability	Complaints or grievances referencing AI advice; AI-related adverse-event reports; and completeness of model, platform, timestamp, and data-source information available for review
Attention gradient and equity	Differences in clinician engagement time, documentation, follow-up, or escalation between patients with and without AI summaries; and differences stratified by language, socioeconomic status, age, disability, rurality, or digital literacy
Documentation burden	Documentation time per AI-related event, length of AI-related documentation, and clinician-reported burden of AI verification and arbitration

a Markers are illustrative and intended to support future empirical evaluation; they are not validated quality measures, performance indicators, clinical protocols, or liability-assignment tools. Their validity, feasibility, equity implications, and risk-stratification sensitivity require further study.

b AI: artificial intelligence.

## Opportunities: Patient Empowerment and the Negotiation of Medical Authority

The potential redistribution of epistemic authority associated with PH-LLM use is not inherently destabilizing. When appropriately governed, these systems may enhance patient participation, improve continuity of care, and support shared decision-making.

### Supporting Patient Participation and Health Literacy

PH-LLMs may strengthen patients’ capacity to participate actively in clinical encounters by supporting personalized sensemaking and preference-sensitive deliberation, although direct evidence for these effects remains limited. By helping users organize symptoms, contextualize home-monitoring data such as weight, blood pressure, glucose, sleep, and activity, and identify questions or concerns for discussion, these systems may enable patients to arrive better prepared for shared decision-making [11]. These functions should augment patients’ participation in clinical reasoning, not substitute for clinician judgment or validated clinical decision support. PH-LLMs may also support health literacy by translating medical terminology, explaining general health concepts at an appropriate reading level, and helping patients formulate questions about test results, care options, and value-sensitive trade-offs [21,22]. Such support may be valuable for individuals with limited baseline health knowledge or low confidence navigating clinical information. At the same time, without safeguards for accuracy, comprehensibility, cultural appropriateness, and bias, PH-LLMs may amplify misunderstanding or misinformation, particularly among underserved populations, despite appearing empathetic and authoritative [2,23].

### Between-Visit Support and Care Delivery Efficiency

At the care-delivery level, LLM-enabled between-visit support may strengthen continuity in chronic disease self-management and extend informational or behavioral support between encounters [24]. Potential applications include medication adherence reminders, structured symptom tracking, and prompts that help patients recognize when to seek professional input [25]. When integrated into well-designed care pathways, such support may contribute to more organized between-visit communication and improve the triage of urgent portal messages [26].

### Collaborative Mediation and the Reframing of Medical Authority

At the level of clinical encounters, PH-LLMs may support more individualized care discussions by organizing patient-reported outcomes, wearable trends, and prior clinical information, where available, into structured summaries that inform agenda setting and shared decision-making [11,27]. Although these systems are sometimes framed as enabling precision medicine, that characterization should be applied cautiously. Current evidence suggests that these systems are better suited to clinical prioritization and patient preparation than to individualized treatment adjustment, which requires rigorous clinician oversight [2,18,28,29]. In this context, clinician authority may be reframed less as unilateral information control and more as collaborative mediation among clinical evidence, patient goals, and AI-generated narratives [30]. This may create a triadic interaction in which the PH-LLM functions as a shared interpretive object that can structure questions, clarify preferences, and support communication [31].

## Risks: Challenges to Medical Order and Medical Authority

### Pre-Encounter Sensemaking as a Source of Risk

Personal health LLMs introduce risks that extend beyond generic health misinformation [15,32]. Because they can generate coherent, personalized, and action-oriented narratives whose surface fluency may exceed their reasoning reliability, they may shape patients’ interpretations of symptoms, risks, and treatment trade-offs before the clinical encounter [16]. This concern is heightened by current limitations in longitudinal medical reasoning, temporal data integration, distribution shift, and hallucination under realistic data conditions [12-14]. Although upstream sensemaking may improve patient preparedness, plausible but nonvalidated outputs may also lead to unsafe actions, distorted expectations, or erosion of therapeutic alignment [17,18].

### Patient-Facing Risks at the Individual Level

Three patient-facing risks may arise at the individual encounter level, even when the model is not overtly hallucinating. First, PH-LLM outputs are sensitive to how patients formulate questions. Users without medical training may describe symptoms ambiguously or omit key context such as red-flag features, comorbidities, or medication use. The system may then generate confident but inaccurate responses that patients carry into clinical encounters as if they reflect validated clinical reasoning [23]. Second, even factually accurate outputs may be misread as personalized recommendations rather than general information requiring individualized clinical judgment. A correct explanation of glycosylated hemoglobin (HbA_1c_), for example, may be misapplied if a patient interprets a specific value without considering comorbidities, hypoglycemia risk, or treatment goals [19,22]. Third, population-level accuracy does not guarantee individual-level appropriateness. PH-LLM outputs may reflect demographic, linguistic, or socioeconomic biases, particularly for populations underrepresented in training data, so correct general information may be misapplied when a patient’s circumstances differ from the model’s implicit reference case [2,23]. These risks warrant health-literacy adaptation, fairness monitoring, and clinician review of higher-risk advice.

### Equity and Access Risks

Equity concerns arise before any model output is generated. Access to paid or premium PH-LLM services, wearable devices, reliable internet connectivity, compatible smartphones, and the digital literacy needed to critically interpret AI-generated narratives is unevenly distributed across populations [33,34]. The triadic model may therefore initially benefit those already better positioned to monitor, organize, and advocate for their health. This imbalance could create an attention gradient: patients who arrive with detailed AI-generated summaries may receive more clinician attention and individualized discussion, while those without such tools may have fewer opportunities to shape the encounter. PH-LLMs should therefore not be assumed to be equity-enhancing simply because they support personalization; their equity effects may vary according to access, usability, affordability, digital literacy, and the fairness of generated outputs [2,18,33].

### Between-Visit Risks: False Alarms and False Reassurance

Between-visit PH-LLM support may produce opposing failure modes. The same functionality that supports continuity may increase utilization through false alarms or delay care through false reassurance when symptom interpretation, triage advice, or escalation guidance is inaccurate or poorly calibrated to clinical urgency. Access and efficiency gains from PH-LLM–enabled between-visit support should therefore be treated as conditional and context-dependent rather than as inherent effects of deployment [35,36].

### Authority Conflict: When Algorithmic and Clinical Recommendations Diverge

A central risk arises when PH-LLM–generated recommendations contradict clinical guidance. Whereas traditional search engines return ranked links that require users to synthesize and interpret information, PH-LLMs can generate contextually tailored, assertive, and seemingly well-referenced recommendations that patients may interpret as equivalent to, or even superior to, clinician judgment [19,22]. As a hypothetical illustration, a patient with type 2 diabetes may receive an LLM-generated suggestion to intensify basal insulin based on continuous glucose monitor data, while the endocrinologist, aware of recent hypoglycemic episodes not captured in the data stream, advises against intensification. In such a scenario, 2 apparently credible sources offer contradictory guidance without a transparent basis for adjudication. Such conflicts may lead some patients to privilege algorithmic outputs over professional judgment, potentially contributing to unsafe actions, reduced trust, or erosion of the therapeutic alliance [19,20]. Clinicians may also experience professional strain when their expertise is challenged by outputs whose assumptions, data sources, and evidentiary limits are difficult to inspect. These tensions are compounded by technical failure modes, including domain mismatch, where wellness-trained models pathologize normal variation; temporal anchoring errors, where models rely on outdated clinical markers; and hallucination, where fabricated assertions are presented with unwarranted confidence [12-14]. Even when clinicians recognize these limitations, patients may lack the expertise to determine when algorithmic advice has exceeded its evidentiary warrant.

### Fragmentation of Clinical Truth: Competing Narratives Across Platforms

Whereas authority conflict concerns tension between algorithmic and clinical guidance, fragmentation arises when multiple PH-LLMs generate divergent interpretations from partial, heterogeneous, or noninteroperable data sources [33,34]. Consumer-facing systems often operate within proprietary data silos, limiting reconciliation across consumer apps, wearable devices, and clinical records. Patients may therefore receive internally coherent but mutually inconsistent health narratives. Such fragmentation may impair the formation of a coherent personal health narrative and contribute to decision paralysis or motivated reasoning, in which patients favor the most reassuring platform over the most clinically appropriate interpretation [37-39]. When platform design is optimized for user engagement, conversational coaching may include behavioral nudges that are not transparently separable from clinical reasoning, raising concerns about persuasive design and commercial conflict [40,41]. Clinicians may therefore be drawn into epistemic triage: identifying clinically valid elements, correcting algorithmically anchored misunderstandings, and reconciling competing narratives without eroding therapeutic trust.

### Diffusion of Accountability: Who Is Responsible When Harm Occurs?

Diffusion of accountability represents a distinct failure mode that concerns governance rather than information quality alone [2,42]. Conventional clinical care rests on defined structures of responsibility: clinicians are accountable for recommendations within their scope of practice, health systems assume organizational duties of care, and regulators set enforceable standards [43,44]. PH-LLMs complicate this architecture by introducing an external source of personalized, clinically suggestive advice that generates diagnostic-style narratives and action-oriented recommendations without comparable mechanisms for auditability, oversight, or duty assignment [45,46]. When patients act on PH-LLM–mediated advice and harm occurs, responsibility may fragment across patients, clinicians, health systems, developers, platforms, and data intermediaries, particularly when data inputs, model assumptions, update history, and downstream data uses remain difficult to inspect [42,47].

This accountability gap may be reinforced when consumer platforms frame outputs as “informational” in their terms of service while presenting them in a clinical tone and with high confidence [48,49]. Clinicians may be asked to respond to advice they did not generate and cannot meaningfully audit, while health systems may lack visibility into the guidance patients receive between visits. Patients, encountering personalized and continuous recommendations, may reasonably treat these outputs as medically authoritative [18,50]. The result is a mismatch between clinical influence and accountable oversight: patients have limited recourse, clinicians face uncertain professional obligations, and health systems remain exposed to risks from decisions shaped outside institutional governance [42,45,47].

Data-governance opacity further compounds this problem. PH-LLMs may rely on sensitive patient-generated health data, wearable streams, and conversational histories, yet consumer-grade practices may not meet health care–grade expectations for security, use limitation, transparency, and breach response [51]. In a distributed ecosystem of device manufacturers, app developers, model providers, cloud services, and data intermediaries, harms arising from unsafe recommendations, data breaches, or opaque secondary uses may be difficult to attribute and remediate [52].

### Privacy and Data Governance

Privacy and data governance warrant dedicated attention in consumer-facing PH-LLMs because users may disclose sensitive health information through open-ended, longitudinal dialog. Unlike static health websites, PH-LLMs may accumulate multisession narratives involving symptoms, medications, mental health concerns, family history, wearable-device trends, and crisis-related disclosures. The extent of such accumulation depends on system design, user settings, and data-retention practices. Privacy risks, therefore, extend beyond unauthorized disclosure to include secondary use, long-term retention, cross-session profiling, inferred sensitive attributes, and blurred boundaries among safety monitoring, product improvement, commercial optimization, and research use [51-53].

Addressing these risks requires context-sensitive governance. General health questions may be managed through baseline consent and adjustable retention settings, whereas mental health disclosures, crisis-related content, and longitudinal behavioral narratives require stronger safeguards, including data minimization, tiered consent, deletion controls, and restrictions on secondary use, advertising, or model training without appropriate authorization [49,51]. Crisis-related content requires an immediate safety response without consent-related delay, followed by access-restricted retention justified by safety or safeguarding needs [2,18,49,51].

Fairness monitoring should be incorporated without making the routine collection of protected-attribute data the default [33,34,51]. Where such data are used for disparity evaluation, they should be separated from interaction content, analyzed in aggregate, and protected through access controls and independent review where feasible [2,33,49,51]. Youth-facing deployments may require additional safeguards for age-appropriate defaults, confidential help-seeking, and crisis-response pathways consistent with applicable child and adolescent data-protection requirements [54,55].

### Summary of System-Level Risks

Taken together, the system-level risks discussed above are mutually reinforcing: authority conflict may worsen fragmentation, fragmentation may obscure responsibility for downstream harms, and unclear privacy practices may undermine clinical appraisal and accountability. When data sources, retention practices, or platform responsibilities are insufficiently disclosed, clinicians and health systems may struggle to assess patient-facing AI narratives and attribute responsibility when harms occur. Privacy governance should therefore be treated as a core component of PH-LLM clinical governance rather than as a peripheral compliance issue.

## Clinical Governance: Managing the Shift and Its Implications

The governance challenge posed by PH-LLMs is not one of comprehensive exclusion but rather of structured clinical integration. Given their increasing availability in consumer-facing health and wellness contexts [50], the comprehensive prohibition of patient-facing AI tools may be neither feasible nor desirable in many settings. Clinical governance should, therefore, focus on making AI-generated narratives visible, assessable, negotiable, and reversible within the professional encounter.

### Limitations of Existing Frameworks

Current regulatory and ethical frameworks for AI in health, including those addressing risk classification, safety assurance, and ethics governance, provide important but incomplete foundations [2,48,56,57]. These frameworks primarily emphasize upstream safety assurance, developer responsibilities, and postdeployment monitoring. They offer limited guidance for situations in which AI-generated narratives originating outside institutional boundaries intersect with clinician judgment, workflow constraints, and shared decision-making. In particular, they do not adequately address: (1) how clinicians should evaluate the credibility of AI outputs generated outside institutional oversight; (2) how conflicts between algorithmic and clinical recommendations should be mediated in practice; and (3) how accountability should be documented when AI advice shapes, but does not determine, clinical decisions. These gaps call for a point-of-care governance model that complements product-level certification with encounter-level governance of patient-brought AI advice.

### A 3-Layer Clinical Governance Framework for PH-LLMs

#### Framework Development

**Figure 2.**
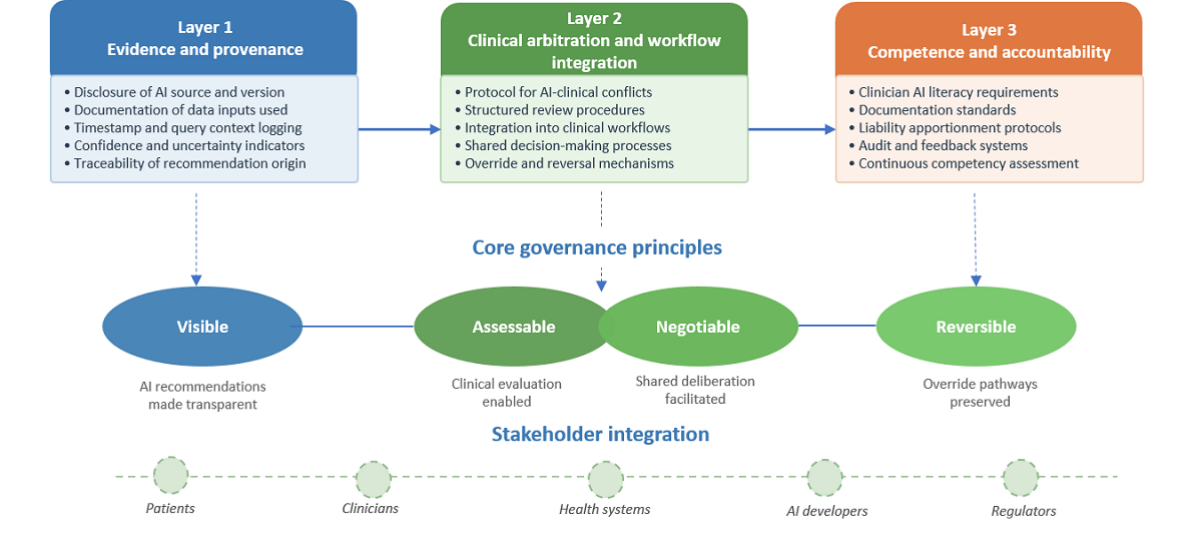
Three-layer clinical governance framework for patient-brought PH-LLM narratives. The framework comprises 3 interdependent layers. Layer 1, evidence and provenance, makes AI-generated narratives legible by identifying the model or platform, data sources, timestamps, uncertainty signals, and privacy-relevant data-use conditions. Layer 2, clinical arbitration and workflow integration, provides a risk-stratified process for determining whether AI advice can be addressed through routine counseling, clinician review, same-day escalation, or specialist input. Layer 3, competence and accountability, clarifies communication skills, documentation expectations, verification duties, and responsibility boundaries among patients, clinicians, institutions, developers, and platforms. Together, these layers are intended to make patient-facing AI narratives visible, assessable, negotiable, privacy-aware, proportionately documented, and accountable within routine clinical workflows. AI: artificial intelligence; PH-LLM: personal health large language model.

To address these gaps, we propose a 3-layer clinical governance framework comprising interdependent layers: (1) evidence and provenance, (2) clinical arbitration and workflow integration, and (3) competence and accountability (Figure 2). The framework was developed through a viewpoint synthesis appropriate to this article type, rather than a systematic review. The synthesis drew on existing AI governance literature [2,18,56-58], digital health and patient-generated health data literature [33,34,51], conceptual frameworks on clinician-patient-AI interaction [30,31], and published literature and time-bound examples of current and emerging consumer-facing PH-LLM use. These inputs were synthesized to identify recurring governance problems, organize them into a 3-layer framework, and propose candidate observable markers for future empirical evaluation (Table 1). The framework is intended to guide the structured integration of PH-LLM narratives into professional practice while safeguarding clinical safety, institutional accountability, and the therapeutic alliance.

#### Layer 1: Evidence and Provenance

A foundational requirement for clinically consequential AI-generated recommendations entering clinical discussion is epistemic legibility: clinicians cannot meaningfully evaluate or negotiate AI advice unless its evidentiary basis is sufficiently visible for clinical appraisal [56,57]. This requirement should be understood as a governance standard for PH-LLMs seeking institutional integration or trusted-use designation, not as a transparency obligation that frontline clinicians can impose on proprietary platforms during routine encounters. For clinically consequential AI outputs, developers and platforms should disclose: (1) model identity and version; (2) data sources, such as wearable data, self-reported symptoms, user-connected personal health records, or user-entered information; (3) temporal anchoring, including timestamps and data windows for key variables; and (4) uncertainty signals distinguishing measured data from inferred or extrapolated conclusions [59]. For example, a patient with hypertension may present a PH-LLM summary concluding that “blood pressure control has worsened” and recommending treatment intensification. A layer 1 provenance check would establish whether this conclusion was based on validated home measurements, wearable estimates, manual entries, or a trend inferred from incomplete data, together with the relevant dates and measurement window. Readings that are recent, consistently elevated, and concordant with the clinical record may inform discussion as patient-provided context; an unclear data source would require confirmation through standard clinical assessment before being treated as clinically actionable evidence.

When provenance is unavailable, clinicians should treat the output as patient-reported contextual information rather than independently verifiable clinical evidence. Clinicians should check clinically consequential claims against the medical record, current clinical findings, guideline-based care, and professional judgment. At the institutional level, health systems may require provenance disclosure, evidence links, audit logs, and data-source labeling as conditions for procurement, formal EHR integration, preferred app lists, or partnerships with commercial PH-LLM vendors [57,58]. Regulators, professional societies, payers, and accreditation bodies may be better positioned to establish enforceable transparency requirements for consumer-facing systems that generate clinically influential recommendations. In practice, full provenance disclosure may remain limited for many consumer-facing PH-LLMs in the near term. Layer 1 should therefore be understood as an aspirational governance standard directed primarily at developers, institutions, and regulators, while layer 2 provides the operational mechanism for handling AI-generated advice at the point of care when provenance is incomplete or unavailable.

#### Layer 2: Clinical Arbitration and Workflow Integration

Governance must extend beyond transparency to specify how AI-generated advice is handled within clinical workflows. We propose a risk-stratified intake process in which AI-generated advice is categorized as follows: (1) lifestyle and health-promotion guidance, generally low risk; (2) disease-management, medication-related, diagnostic-interpretation, or monitoring advice, moderate to high risk; or (3) safety-critical alerts requiring immediate escalation [17,30,31,48]. To remain feasible, this process should not require extensive documentation for every AI-related discussion. Low-risk advice may be addressed through ordinary clinical counseling and documented, if needed, within the standard encounter note. More structured arbitration should be reserved for cases in which AI-generated advice materially conflicts with clinical judgment, guideline-based care, medication decisions, diagnostic interpretation, or escalation thresholds. Safety-critical alerts, including indications of acute risk such as suicidal ideation, cardiac symptoms, or severe medication interactions, should trigger immediate clinical escalation through established institutional pathways rather than routine arbitration.

Workflow integration should also protect equity in clinical attention because patient-generated health data, wearable devices, and commercial digital tools are unevenly available across populations [33,34]. Triage should remain grounded in clinical acuity, patient vulnerability, and care needs rather than the presence or sophistication of an AI-generated summary. Health systems may reduce inequity through standardized previsit symptom forms, nonproprietary patient-reported outcome tools, interpreter-accessible digital supports, and staff-assisted intake, helping prevent PH-LLM use from becoming an informal gateway to more attentive care.

When AI-generated advice falls into moderate-risk or high-risk categories, documentation should be embedded into existing workflows rather than added as a separate administrative layer. Practical mechanisms may include previsit intake questions, patient-uploaded screenshots with timestamps and data windows where appropriate, EHR smart phrases, check-box templates, and team-based triage before clinician review [34,48,51]. For moderate-risk or high-risk advice, including safety-critical items, minimum documentation should capture the clinically relevant AI-generated claim or recommendation, the reason for accepting, modifying, or rejecting it, any patient preference that materially affected the decision, and the final shared plan. This approach supports accountable reasoning without converting AI-related disagreement into an excessive administrative burden. To make this layer actionable, Table 2 provides an illustrative PH-LLM intake template organized around 4 stages: patient-provided information, staff triage, minimum documentation, and escalation triggers. The template is risk-calibrated and intended for local adaptation rather than as a validated protocol.

**Table 2.** Illustrative personal health large language models (PH-LLM) intake template for receiving, triaging, documenting, and escalating patient-brought artificial intelligence (AI) advice in routine encounters.^a^

Operational stage	Recommended elements	Example operationalization
Patient-provided information	Screenshot or text of the AI output; platform or model name if visible; date and time; relevant data window, such as 7 or 30-day wearable trends; patient’s reason for raising the advice	Portal upload, check-in photograph, or brief intake note, such as, “I asked [PH-LLM] on [date] about [concern].”
Staff triage	Apply the layer 2 risk-tier classification to incoming AI advice: low risk, moderate or high risk, or safety-critical	EHR^b^ smart phrase or intake prompt with risk-tier checkbox; moderate-risk or high-risk and safety-critical items routed for clinician review
Minimum documentation	For moderate-risk, high-risk, and safety-critical advice: AI-generated claim, clinician rationale for accepting, modifying, or rejecting it, patient preference if material, and final shared plan^c^	Embedded EHR smart phrase or check-box template, rather than a separate administrative form
Escalation triggers	Same-day review, specialist consultation, or safety pathway when AI advice may affect medication, diagnostic interpretation, or escalation decisions; conflicts with recommended treatment; poses substantial foreseeable harm; or shows recurrent unsupported reliance	“AI-Flag” routed to clinician inbox; same-day phone or telehealth contact for safety-critical items; specialist consultation or safety event report for clinically consequential or recurrent conflicts

a This template is illustrative and risk-calibrated; fields, thresholds, and pathways should be locally adapted and empirically evaluated.

b EHR: electronic health record.

c Low-risk advice does not require structured documentation and may be addressed within the standard counseling note when clinically relevant.

This layer should also include reversibility mechanisms. Reversibility does not mean overriding competent patient autonomy or prohibiting PH-LLM use; rather, it means revisiting, contextualizing, or limiting the clinical influence of AI advice that is unsupported, outdated, unsafe, or inconsistent with the patient’s broader clinical context. Low-risk lifestyle guidance may be managed through shared decision-making, risk communication, monitoring, and follow-up, whereas clinically consequential or safety-critical conflicts may require secondary review, specialist consultation, or institutional escalation [17,20,30]. For external consumer-facing systems outside institutional control, the absence of provenance limits the evidentiary weight of the output, and clinically consequential recommendations should not be relied on without independent verification. This approach reframes conflict not merely as an error to be corrected but as a managed negotiation within accountable care, while avoiding a paternalistic model in which clinician authority automatically displaces AI-informed patient preferences.

#### Layer 3: Competence and Accountability

Effective governance requires new competencies and clarified responsibility boundaries. For clinicians, this includes AI-related clinical communication competence: the ability to redirect AI-driven disagreement away from comparative intelligence, such as “AI versus clinician,” toward evidence quality, risk assessment, patient values, and verifiable outcomes [53]. Clinicians should also recognize automation bias in patients and use concise, nonconfrontational strategies to restore epistemic balance without undermining patient agency [17,19,20]. Patients, in turn, require basic AI literacy. Health systems should provide standardized guidance, analogous to medication education, on red-flag symptoms, data currency, and documentation practices, including screenshots with timestamps and relevant data windows [17,20,53]. At the institutional level, health systems should establish documentation standards, escalation pathways, audit mechanisms, and adverse-event review processes for AI-influenced clinical decisions [42,46,48].

Accountability should be allocated according to control and role. Clinicians should not be held responsible for the internal logic, model design, data-processing errors, or algorithmic failures of PH-LLMs they did not develop, deploy, control, or audit [42,45,47]. However, when AI-generated advice is presented during care and materially influences diagnosis, treatment, escalation, or shared decision-making, clinicians may retain a professional duty to evaluate clinically consequential claims through reasonable, risk-proportionate verification [30,43,47,48]. Developers and platforms remain responsible for product design, provenance, privacy practices, and algorithmic behavior; institutions are responsible for workflow standards, escalation pathways, documentation expectations, and audit mechanisms; and clinicians are responsible for responding reasonably to AI-influenced claims that become visible and clinically relevant [42,45-47]. For example, a patient with chronic kidney disease may report a PH-LLM recommendation for frequent over-the-counter nonsteroidal anti-inflammatory drug use. Under layer 3, the clinician is not responsible for the model’s reasoning or design, but has a risk-proportionate responsibility to evaluate a claim that is clinically visible and consequential. This would include verifying renal function and medications, explaining the risk, documenting the decision to reject or modify the advice, and offering a safer alternative. Recurrent or foreseeably harmful recommendations could be escalated through medication-safety review, a digital health governance committee, or vendor-reporting channels.

This distinction helps clarify the liability gray area. Documented verification should not be understood as automatically transferring liability for opaque external systems to clinicians; rather, it creates an auditable record showing that clinically consequential AI advice was accepted, modified, or rejected on the basis of clinical evidence, patient context, and professional judgment [43,47,48]. Conversely, ignoring a clearly safety-relevant AI-generated claim or relying on it without appropriate verification may increase professional or institutional risk under existing negligence standards [43,44,47]. Clearer institutional policies and regulatory guidance are therefore needed to distinguish product-level accountability from clinician-level duties of reasonable verification in triadic clinician-patient-PH-LLM interactions [31,42,44-48].

### Toward Empirical Evaluation: Observable Markers

Key concepts in this viewpoint, including epistemic authority, negotiated authority, authority conflict, fragmentation of clinical truth, and diffusion of accountability, describe potential sociotechnical implications associated with PH-LLM use in clinical encounters [30,42,45,53]. To support future empirical examination, Table 1 maps these concepts to candidate observable markers. These markers are illustrative rather than validated performance metrics, quality indicators, clinical protocols, or liability-assignment tools; their validity, feasibility, equity implications, and sensitivity to clinical risk stratification require further study [34,51]. They may be operationalized through structured clinical documentation, EHR smart phrases, encounter recording or time-and-motion studies where ethically approved and consented, patient-reported measures, equity-stratified visit analytics, and patient safety event reporting systems [34,46,48]. Their use should be proportionate to clinical risk and should avoid adding excessive documentation burden or creating incentives to privilege AI-generated summaries over clinically similar concerns raised without AI assistance.

## Limitations

This viewpoint has several limitations. First, it is conceptual and governance-oriented; the proposed 3-layer framework, intake template (Table 2), and observable markers (Table 1) are illustrative, have not been empirically validated, and should not be used as performance measures, quality indicators, or clinical protocols without further validation. Second, as a viewpoint study, the literature synthesis is narrative and conceptually motivated rather than systematic; readers seeking exhaustive evidence appraisal on specific PH-LLM capabilities or harms should consult systematic reviews, benchmark studies, and future empirical evaluations as the evidence base matures. Third, the framework is informed primarily by US clinical, regulatory, and liability contexts, and adaptation may be required for other health systems, financing and payment models, professional norms, and legal frameworks. Fourth, the framework focuses primarily on patient-facing conversational PH-LLM narratives brought into clinical encounters and may require adaptation for fully multimodal systems, EHR-integrated tools, or institutionally deployed clinical decision-support systems. Fifth, the framework addresses downstream clinical governance of PH-LLM advice in encounters rather than upstream technical infrastructure of PH-LLM deployment. Service-level and infrastructure factors, such as latency, uptime, update frequency, runtime reconfiguration, and quality-of-service management, may affect deployed PH-LLM reliability, but detailed infrastructure governance is outside the scope of this framework [60,61]. Finally, the consumer-facing product examples cited, such as ChatGPT Health and Fitbit’s Gemini-powered health coach, are time-bound illustrations of an evolving ecosystem; their features, availability, and access conditions remain subject to change. Future work should prospectively evaluate the feasibility, documentation burden, equity effects, clinical safety impact, and acceptability of the proposed governance components across diverse care settings.

## Conclusions

Ungoverned PH-LLMs may destabilize how clinical authority is negotiated, fragment therapeutic relationships, and diffuse accountability when patient-facing AI narratives enter care. When governed through the proposed 3-layer framework, these narratives may instead be incorporated as supporting infrastructure for safer, person-centered, longitudinal care. The task is not to exclude AI from clinical relationships, but to render AI-generated narratives clinically visible, negotiable, verifiable, and accountable within structured clinical governance.

## Abbreviations

AI: artificial intelligence
EHR: electronic health record
HbA_1c_: glycosylated hemoglobin
LLM: large language model
PH-LLM: personal health large language model
